# Use of 21-gene recurrence score assay to individualize adjuvant chemotherapy recommendations in ER+/HER2− node positive breast cancer—A National Cancer Database study

**DOI:** 10.1038/s41523-017-0044-4

**Published:** 2017-10-19

**Authors:** Prema P. Peethambaram, Tanya L. Hoskin, Courtney N. Day, Matthew P. Goetz, Elizabeth B. Habermann, Judy C. Boughey

**Affiliations:** 1 Medical Oncology, Mayo Clinic, Rochester, MN USA; 2 Health Sciences Research, Mayo Clinic, Rochester, MN USA; 3 Health Care Policy and Research and Robert D. and Patricia E. Kern Center for the Science of Health Care Delivery, Mayo Clinic, Rochester, MN USA; 4 Surgery, Mayo Clinic, Rochester, MN USA

## Abstract

The 21-gene Recurrence Score (RS) assay is prognostic and predictive of adjuvant chemotherapy benefit in node positive (N+) breast cancer (BC). We sought to evaluate use patterns of RS assay in N+, ER+/HER2− BC and the impact of RS on recommendations for adjuvant chemotherapy. Patients with T1-T4c,N1mi-N3, ER+/HER2− BC diagnosed 2010–2013 in the National Cancer Database were analyzed. Multivariable logistic regression assessed factors influencing RS testing and chemotherapy recommendations based on RS. Among 72,897 patients, RS was obtained in 20.6%, increasing from 15.0% in 2010 to 24.5% in 2013 (*p* < 0.001). RS testing was most common in N1mi (43.7%) followed by N1 (22.1%) and rare in N2/N3 (3.3%). Of the 12,536 with quantitative RS results, 61.1% were low RS, 32.3% intermediate RS and 6.6% high RS. Chemotherapy was recommended less frequently in patients with RS testing (50.4%) vs. those not tested (81.0%, *p* < 0.001). In N1mi/N1 patients, chemotherapy recommendation varied by RS; however, in N2/N3 patients, chemotherapy was recommended in the majority (70.9–87.5%) regardless of RS. Most patients (>85%) with RS ≥ 26 were recommended chemotherapy regardless of nodal stage. For patients with RS < 26, chemotherapy recommendations increased with higher N and T stage, grade, and younger age (*p* < 0.001). Histology was not associated with chemotherapy recommendation in any RS subset. The RS assay is frequently and increasingly being used for decision making in node positive ER+/HER2− breast cancer patients and its use is associated with lower rates of adjuvant chemotherapy.

## Diagnostics: more US women getting gene test, avoiding chemo

Use of the 21-gene recurrence score (RS) assay is going up, helping many women avoid the burden of chemotherapy. Prema Peethambaram and colleagues from the Mayo Clinic in Rochester, Minnesota, USA, analyzed the records of around 73,000 women diagnosed over 4 years in the United States with breast tumors that have estrogen receptors, but no HER2 protein, and have spread to the lymph nodes. They found that RS testing increased from 15% in 2010 to 24.5% in 2013. Among those who received the test, also known as Oncotype DX, only 50% underwent chemotherapy (in addition to endocrine therapy) after surgery, compared to 81% of women not tested. The findings suggest that RS testing can help identify low-risk individuals who stand little to gain from adjuvant chemotherapy. Large prospective studies are ongoing to validate this conclusion.

## Introduction

For the treatment of estrogen receptor (ER) positive breast cancer, adjuvant endocrine therapy provides the greatest benefit in terms of reduction in distant recurrence and breast cancer mortality. For example, it is estimated that during the first 4 years of adjuvant endocrine treatment aromatase inhibitors reduce the risk of recurrence by 63% and breast cancer mortality by 44% compared to no endocrine therapy and that this same regimen provided a “carryover” effect, with an additional 37% reduction in risk in years 5–9.^1^ More recently, extended adjuvant endocrine therapy from 5–10 years has resulted in further reductions in recurrence.^2–4^ Despite this substantial benefit of endocrine therapy, chemotherapy is still recommended for women with ER+ node positive breast cancer, based on a meta-analysis of adjuvant chemotherapy studies demonstrating an approximate 22% reduction in breast cancer mortality for the use of adjuvant polychemotherapy for postmenopausal women.^5^

With advances in understanding of tumor biology, guidelines for the use of adjuvant chemotherapy have shifted away from a sole focus on anatomic staging to incorporating biological factors to help guide systemic treatment recommendations. Several different commercially available multi-gene assays such as the 21 gene Recurrence Score assay, Oncotype DX),^6^ 70 gene assay (MammaPrint),^7^ Prosigna based on PAM50,^8–11^ 12 gene assay (Endopredict)^12,13^ and Breast Cancer Index^14,15^ are available and these provide prognostic information independent of standard clinical and pathological factors. The 21 gene recurrence score (RS)^6,16^ has been determined to be prognostic in both node negative and node positive ER+/HER2− breast cancer.^16–20^ Additionally, the RS has been studied in secondary analysis of prospective clinical trials which tested the benefit of adjuvant chemotherapy and provides limited evidence that RS is predictive of chemotherapy benefit in node positive ER+/HER2− breast cancer patients.^17–22^ Based on these studies, NCCN guidelines recommends the use of the RS in decision making in the setting of both node negative and node positive (1–3 lymph nodes) ER+ breast cancer. In contrast, the American Society of Clinical Oncology guidelines state that the clinician should not use the 21-gene RS to guide therapy in node positive ER+/HER2− breast cancer.^23^ While prospective clinical trials are ongoing to answer this question, these disparate recommendations may lead to confusion among patients and providers and thus impact reimbursement by payers.^22^

Given the emerging data for the role of the RS, as well as the recent discrepant guidelines, we sought to assess recent practice patterns to evaluate both the factors influencing RS testing and the impact of RS results on adjuvant chemotherapy recommendations in node positive, ER+/HER2− breast cancer patients using the National Cancer Database (NCDB).

## Results

**Fig. 1:**
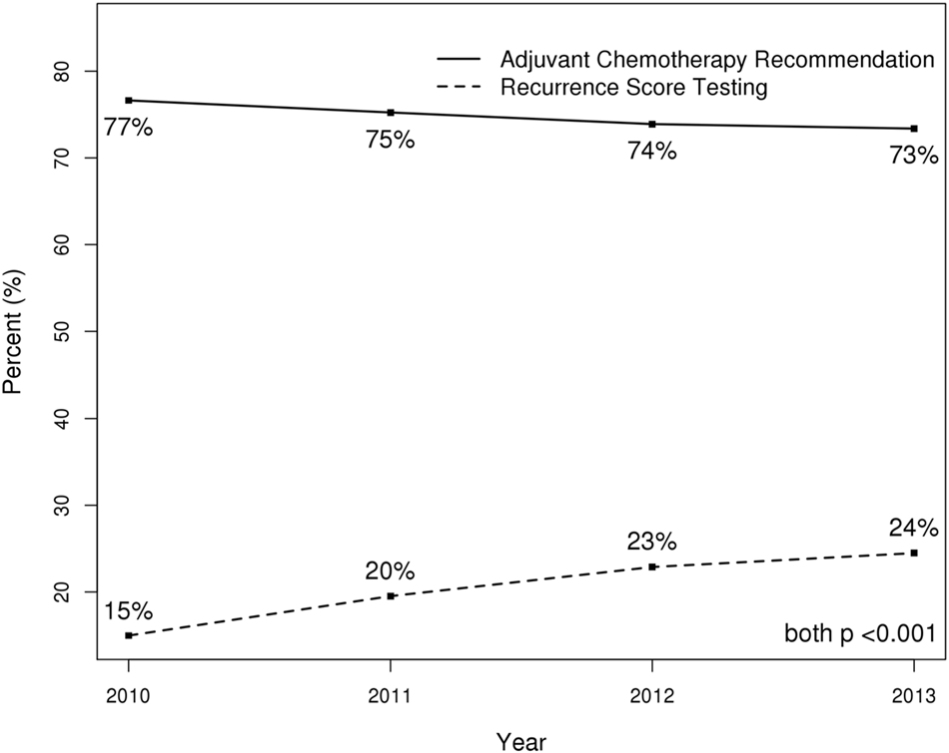
Line chart demonstrating change over time in the percentage of female ER+/HER2− node positive patients recommended to undergo adjuvant chemotherapy and change over time in the percentage with RS testing

Of 72,897 women with T1-T4c, N1mi-N3, ER positive, HER2-negative breast cancer, RS was obtained in 15,028 (20.6%). The demographic and clinical characteristics of the entire cohort are summarized in Table 1. Use of RS testing increased significantly over the time period studied from 15.0% of patients tested in 2010 to 24.5% in 2013 (*p* < 0.001), and the recommendation for adjuvant chemotherapy showed a small but statistically significant decrease over the same period (*p* < 0.001) (Fig. 1).

**Table 1:** Univariate and multivariable analysis of factors associated with use of RS testing

		RS testing		Univariate		Multivariable	
Variable	Total *N* = 72,897 N (%)	No *N* = 57,869 N (%)	Yes *N* = 15,028 N (%)	Odds Ratio (95% CI)	*P*-value	Odds Ratio (95% CI)	*P*-value
Age category					Overall trend *p* < 0.001		Overall trend *p* = 0.35
<40	4052 (5.6%)	3601 (88.9%)	451 (11.1%)	0.45 (0.41, 0.50)	<0.001	0.63 (0.56, 0.71)	<0.001
40–49	14,727 (20.2%)	12,042 (81.8%)	2685 (18.2%)	0.80 (0.76, 0.85)	<0.001	0.79 (0.74, 0.83)	<0.001
50–59	19,858 (27.2%)	15,547 (78.3%)	4311 (21.7%)	1.0 reference		1.0 reference	
60–69	18,889 (25.9%)	14,104 (74.7%)	4785 (25.3%)	1.22 (1.17, 1.28)	<0.001	1.23 (1.17, 1.30)	<0.001
70–79	10,554 (14.5%)	8086 (76.6%)	2468 (23.4%)	1.10 (1.04, 1.16)	<0.001	1.12 (1.04, 1.21)	0.003
80+	4817 (6.6%)	4489 (93.2%)	328 (6.8%)	0.26 (0.23, 0.30)	<0.001	0.27 (0.24, 0.31)	<0.001
Race							
Black	7856 (10.8%)	6583 (83.8%)	1273 (16.2%)	0.71 (0.67, 0.76)	<0.001	0.81 (0.76, 0.87)	<0.001
Other/unknown	3615 (5.0%)	2939 (81.3%)	676 (18.7%)	0.85 (0.78, 0.93)	<0.001	0.88 (0.80, 0.96)	0.005
White	61,426 (84.3%)	48,347 (78.7%)	13,079 (21.3%)	1.0 reference		1.0 reference	
Charlson–Deyo score					Overall trend *p* < 0.001		Overall trend *p* = 0.12
0	60,427 (82.9%)	47,783 (79.1%)	12,644 (20.9%)	1.0 reference		1.0 reference	
1	10,205 (14.0%)	8233 (80.7%)	1972 (19.3%)	0.91 (0.86, 0.95)	<0.001	0.93 (0.87, 0.98)	0.01
2+	2265 (3.1%)	1853 (81.8%)	412 (18.2%)	0.84 (0.75, 0.94)	<0.001	0.91 (0.80, 1.02)	0.10
Primary payor							
Private insurance/managed care	40,912 (56.1%)	32,046 (78.3%)	8866 (21.7%)	1.0 reference		1.0 reference	
Medicaid/ not insured	7542 (10.3%)	6402 (84.9%)	1140 (15.1%)	0.64 (0.60, 0.69)	<0.001	0.77 (0.72, 0.83)	<0.001
Medicare	22,729 (31.2%)	18,037 (79.4%)	4692 (20.6%)	0.94 (0.90, 0.98)	0.002	0.97 (0.92, 1.03)	0.35
Other government	863 (1.2%)	701 (81.2%)	162 (18.8%)	0.84 (0.70, 0.99)	0.04	0.88 (0.73, 1.06)	0.19
Insurance status unknown	851 (1.2%)	683 (80.3%)	168 (19.7%)	0.89 (0.75, 1.05)	0.18	0.93 (0.77, 1.11)	0.41
Year of diagnosis					Overall trend *p* < 0.001		Overall trend *p* < 0.001
2010	16,819 (23.1%)	14,301 (85.0%)	2518 (15.0%)	1.0 reference		1.0 reference	
2011	18,496 (25.4%)	14,886 (80.5%)	3610 (19.5%)	1.38 (1.30, 1.46)	<0.001	1.39 (1.31, 1.47)	<0.001
2012	18,779 (25.8%)	14,482 (77.1%)	4297 (22.9%)	1.69 (1.60, 1.78)	<0.001	1.74 (1.64, 1.85)	<0.001
2013	18,803 (25.8%)	14,200 (75.5%)	4603 (24.5%)	1.84 (1.74, 1.94)	<0.001	1.91 (1.80, 2.02)	<0.001
Facility type							
Comprehensive community cancer program	34,845 (47.8%)	27,622 (79.3%)	7223 (20.7%)	1.20 (1.13, 1.28)	<0.001	1.16 (1.09, 1.25)	<0.001
Academic/research program	21,024 (28.8%)	16,101 (76.6%)	4923 (23.4%)	1.41 (1.32, 1.50)	<0.001	1.41 (1.31, 1.51)	<0.001
Integrated network cancer program/other	5149 (7.1%)	4115 (79.9%)	1034 (20.1%)	1.16 (1.06, 1.26)	0.001	1.08 (0.98, 1.19)	0.13
Facility type unknown	4052 (5.6%)	3601 (88.9%)	451 (11.1%)	0.58 (0.51, 0.65)	<0.001	–	–
Community cancer program	7827 (10.7%)	6430 (82.2%)	1397 (17.8%)	1.0 reference		1.0 reference	
Pathologic T stage					Overall Trend *p* < 0.001		Overall Trend *p* < 0.001
T1	32,178 (44.1%)	22,860 (71.0%)	9318 (29.0%)	1.0 reference		1.0 reference	
T2	32,714 (44.9%)	27,470 (84.0%)	5244 (16.0%)	0.47 (0.45, 0.49)	<0.001	0.67 (0.65, 0.70)	<0.001
T3/T4 a-c	8005 (11.0%)	7539 (94.2%)	466 (5.8%)	0.15 (0.14, 0.17)	<0.001	0.30 (0.27, 0.34)	<0.001
Pathologic N stage					Overall Trend *p* < 0.001		Overall Trend *p* < 0.001
N1mi	10,773 (14.8%)	6068 (56.3%)	4705 (43.7%)	1.0 reference		1.0 reference	
N1	44,126 (60.5%)	34,391 (77.9%)	9735 (22.1%)	0.37 (0.35, 0.38)	<0.001	0.40 (0.38, 0.42)	<0.001
N2+	17,998 (24.7%)	17,410 (96.7%)	588 (3.3%)	0.04 (0.04, 0.05)	<0.001	0.06 (0.05, 0.06)	<0.001
Grade					Overall Trend *p* < 0.001		Overall Trend *p* < 0.001
Well differentiated	13,127 (18.0%)	9306 (70.9%)	3821 (29.1%)	1.0 reference		1.0 reference	
Moderately differentiated	37,892 (52.0%)	29,687 (78.3%)	8205 (21.7%)	0.67 (0.64, 0.70)	<0.001	0.83 (0.79, 0.87)	<0.001
Poorly differentiated/Undifferentiated	18,037 (24.7%)	15,756 (87.4%)	2281 (12.6%)	0.35 (0.33, 0.37)	<0.001	0.54 (0.50, 0.57)	<0.001
Cell type undetermined	3841 (5.3%)	3120 (81.2%)	721 (18.8%)	0.56 (0.51, 0.62)	<0.001	0.67 (0.61, 0.74)	<0.001
ILC histology							
Yes	10,265 (14.1%)	8441 (82.2%)	1824 (17.8%)	1.0 reference		1.0 reference	
No	62,632 (85.9%)	49,428 (78.9%)	13,204 (21.1%)	1.24 (1.17, 1.30)	<0.001	0.94 (0.88, 1.0)	0.035
Progesterone receptor (PR) status							
Negative	7396 (10.1%)	6302 (85.2%)	1094 (14.8%)	1.0 reference		1.0 reference	
Positive	65,390 (89.7%)	51,473 (78.7%)	13,917 (21.3%)	1.56 (1.46, 1.67)	<0.001	1.27 (1.18, 1.37)	<0.001
Unknown	111 (0.2%)	94 (84.7%)	17 (15.3%)	1.04 (0.62, 1.75)	<0.001	1.0 (0.57, 1.76)	>0.99

### Patient demographic factors impacting use of RS testing

The median age of patients who underwent RS testing was 60.0 (interquartile range (IQR) 51.0 to 67.0) years which was slightly higher than the median age of patients who did not undergo RS testing (58.0 years, IQR 49.0 to 68.0 years, *p* < 0.001). Analyzing by age group, RS testing was most commonly utilized in patients aged 50–79 (23.5%) and less common at the extremes of age with 6.8% of patients age 80+ and 11.1% of patients age < 40 undergoing testing. RS testing was more likely to be performed in white patients than black patients (21.3 vs. 16.2%, *p* < 0.001). RS testing was more commonly obtained in patients who had private insurance/managed care (21.7%) or Medicare (20.6%), than among those with Medicaid or no insurance (15.1%, *p* < 0.001). RS testing was more commonly utilized in academic/research programs (23.4%) and comprehensive community cancer programs (20.7%) compared to community cancer programs (17.8%, *p* < 0.001).

### Distribution of recurrence scores

Of the 15,028 patients who underwent RS testing the numeric score result was unknown in 2492 (16.6%). Of the 12,536 with quantitative RS results, 61.1% were low RS, 32.3% intermediate RS and 6.6% high RS. The median RS was 15.0 (IQR 11.0, 21.0) in this patient cohort.

### Tumor factors influencing RS testing

RS testing rate was higher in patients with pT1 disease (29.0%) and decreased with advancing tumor stage (Table 1). A similar trend was observed for node stage as 43.7% of pN1mi patients, 22.1% of pN1 patients, and only 3.3% of pN2/pN3 patients underwent RS testing. RS assay was more frequently obtained in patients with well differentiated and moderately-differentiated tumors than poorly-differentiated disease (*p* < 0.001). RS assay was slightly less commonly ordered in invasive lobular carcinoma (17.8%) than other histologies (21.1%, *p* < 0.001).

Overall, 65,390 (89.7%) of the N+ ER+ were also progesterone receptor (PR) positive. Of the 15,028 patients with RS testing performed, PR was positive in 13,917 (92.6%), negative in 1094 (7.3%) patients, and unknown in 17 (0.1%). RS testing was more commonly performed in PR positive patients than in PR negative patients (21.3% of PR positive patients compared to 14.8% of PR negative patients (*p* < 0.001) underwent RS testing).

### Multivariable analysis of factors impacting use of RS testing

We next fit a multivariable model looking at the factors impacting the use of RS testing and their adjusted ORs (Table 1). On multivariable analysis patient factors that remained significantly associated with lower use of RS were extremes of age, black race, and Medicaid and non-insured patients (all *p* < 0.001). Tumor factors associated with lower use of RS testing on multivariable analysis included more advanced nodal disease, larger tumor size, PR negative, and poorly-differentiated tumors. After adjustment for other factors, invasive lobular histology was no longer significantly associated with decreased likelihood of testing.

### Impact of RS testing on recommending adjuvant chemotherapy

In patients who underwent RS testing, adjuvant chemotherapy was recommended for 50.4% whereas in patients who did not undergo RS testing, adjuvant chemotherapy was recommended for 81.0% (*p* < 0.001). After adjusting for patient and clinical factors, adjuvant chemotherapy recommendation remained less likely in those who underwent RS testing (OR: 0.25, 95% CI: 0.24–0.26, *p* < 0.001).

Of the patients in the low RS group, 35.6% were recommended adjuvant chemotherapy whereas in the high RS group, 93.2% of patients were recommended adjuvant chemotherapy. In patients with intermediate RS, there was a significant difference in adjuvant chemotherapy recommendation between RS of 18–25 where adjuvant chemotherapy was recommended in 66.3% and patients with RS of 26–30 where adjuvant chemotherapy was recommended in 85.3% of patients (*p* < 0.001).

Some patients who were recommended chemotherapy did not go on to receive chemotherapy for a variety of reasons such as patient refusal, death prior to starting chemotherapy, or for unknown reasons. Overall 10.0% of patients were recommended but did not receive chemotherapy. This was higher in those that underwent RS testing (23.6%) than in patients without RS testing (7.8%, *p* < 0.001). The proportion of patients where chemotherapy was recommended by the patient’s physician but not administered was lower in the high RS group at 8.4% (65/770) compared to 34.3% (937/2728) in the low RS patients, suggesting that the RS result also impacted the patient’s decision.

### Pathologic N stage and adjuvant chemotherapy recommendations

**Fig. 2:**
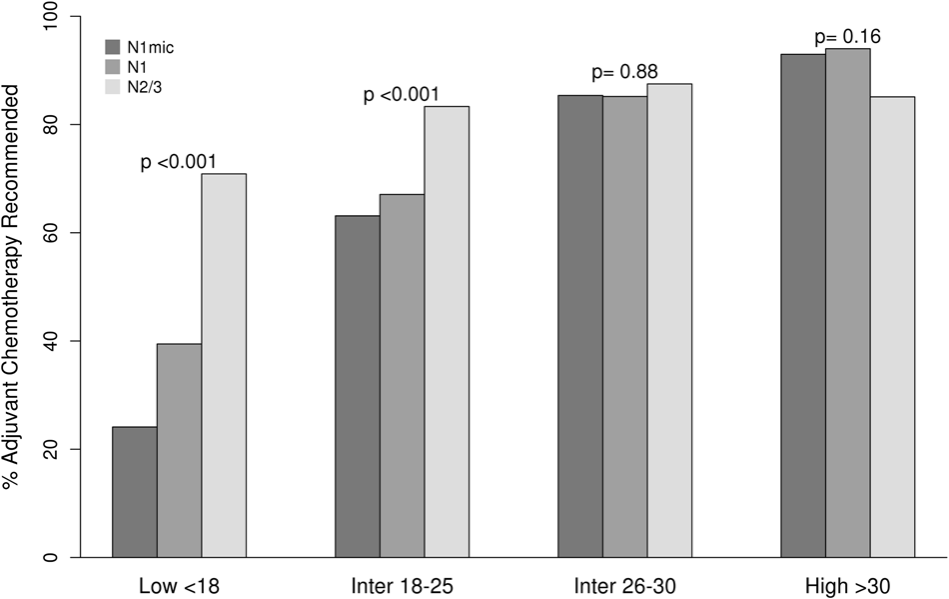
Bar diagram showing percent recommended adjuvant chemotherapy by N stage and RS

Adjuvant chemotherapy was recommended in 85–94% of high RS patients regardless of pathologic nodal stage (Fig. 2). However, in the low RS group a strong association was observed between pathologic nodal stage and the recommendation for adjuvant chemotherapy with 24.1% of N1mi patients and 39.5% of N1 patients being recommended adjuvant chemotherapy compared to 70.9% of N2/N3 patients (*p* < 0.001). A significant increase in the rates of recommendation for adjuvant chemotherapy with increasing pathologic nodal stage was also observed in patients with RS 18–25 with rates of 63.1% (N1mi), 67.1% (N1), and 83.3% (N2/N3), *p* < 0.001, but this pattern was not seen for RS 26–30 where chemotherapy was recommended in 85% of patients with N1mi and N1 disease and in 88% with N2/N3 disease. (Fig. 2).

### Factors impacting recommendations for adjuvant chemotherapy by RS

We proceeded to perform multivariable logistic models to assess factors impacting recommendation for adjuvant chemotherapy stratified by RS testing result (Table 2). A large majority (93%) of patients in the high RS group were recommended to receive adjuvant chemotherapy. Among those with high RS, recommendation for chemotherapy was significantly associated with age and grade, and the patients with the lowest probability of recommendation for adjuvant chemotherapy (predicted probability <80%) despite their high RS were 80+ years of age or 70–79 years with well-differentiated tumors.

**Table 2:** Multivariable analysis of factors that impacted recommendation for adjuvant chemotherapy stratified by RS testing result

	Low < 18		Intermediate 18–25		Intermediate 26–30	
Variable	Odds ratio (95% CI)	*P*-value	Odds ratio (95% CI)	*P*-value	Odds ratio (95% CI)	*P*-value
Age category		Overall trend *p* < 0.001		Overall trend *p* < 0.001		Overall trend *p* < 0.001
<40	3.13 (2.22, 4.41)	<0.001	3.62 (1.90, 6.88)	<0.001	5.20 (0.31, 87.74)	0.25
40–49	1.71 (1.49, 1.97)	<0.001	1.84 (1.43, 2.36)	<0.001	1.86 (0.74, 4.69)	0.19
50–59	1.0 reference		1.0 reference		1.0 reference	
60–69	0.68 (0.60, 0.77)	<0.001	0.66 (0.55, 0.80)	<0.001	0.44 (0.24, 0.81)	0.008
70–79	0.37 (0.31, 0.43)	<0.001	0.31 (0.25, 0.40)	<0.001	0.14 (0.07, 0.26)	<0.001
80+	0.18 (0.12, 0.29)	<0.001	0.10 (0.06, 0.18)	<0.001	0.08 (0.03, 0.23)	<0.001
Charlson–Deyo score		Overall trend *p* = 0.91		Overall trend *p* = 0.008		Overall trend *p* = 0.83
0	1.0 reference		1.0 reference		1.0 reference	
1	0.95 (0.82, 1.10)	0.50	0.78 (0.62, 0.97)	0.02	1.16 (0.63, 2.15)	0.63
2+	1.03 (0.76, 1.39)	0.86	0.69 (0.42, 1.16)	0.16	0.66 (0.25, 1.78)	0.41
Pathologic T stage		Overall trend *p* < 0.001		Overall trend *p* = 0.06		Overall trend *p* = 0.12
T1	1.0 reference		1.0 reference		1.0 reference	
T2	1.33 (1.19, 1.48)	<0.001	1.17 (0.99, 1.38)	0.08	1.27 (0.81, 1.99)	0.30
T3/T4 a-c	2.16 (1.61, 2.89)	<0.001	1.34 (0.81, 2.23)	0.26	3.87 (0.53, 28.33)	0.18
Pathologic N stage		Overall trend *p* < 0.001		Overall trend *p* < 0.001		Overall trend *p* = 0.49
N1mi	1.0 reference		1.0 reference		1.0 reference	
N1	2.25 (2.01, 2.52)	<0.001	1.38 (1.17, 1.63)	<0.001	1.18 (0.74, 1.88)	0.50
N2+	8.93 (6.61, 12.07)	<0.001	3.67 (2.06, 6.54)	<0.001	0.86 (0.22, 3.28)	0.82
Grade		Overall trend *p* < 0.001		Overall trend *p* < 0.001		Overall trend *p* = 0.23
Well differentiated	1.0 reference		1.0 reference		1.0 reference	
Moderately differentiated	1.17 (1.05, 1.32)	0.005	1.48 (1.23, 1.79)	<0.001	1.36 (0.71, 2.62)	0.35
Poorly differentiated/Undifferentiated	1.63 (1.34, 1.98)	<0.001	2.27 (1.75, 2.95)	<0.001	1.61 (0.80, 3.25)	0.18
Cell type undetermined	1.07 (0.84, 1.37)	0.60	1.40 (0.96, 2.05)	0.08	0.53 (0.18, 1.54)	0.24
ILC histology						
Yes	1.0 reference		1.0 reference		1.0 reference	
No	1.08 (0.93, 1.26)	0.30	0.94 (0.74, 1.19)	0.60	0.86 (0.39, 1.88)	0.70

We subsequently focused this analysis on the low and intermediate RS groups. In patients who had a low RS, 35.6% of patients were still recommended to receive adjuvant chemotherapy. Adjuvant chemotherapy was more likely to be recommended in younger patients, those with more advanced nodal stage (OR 2.25 and OR 8.93 for N1 and N2/N3, respectively, each vs. N1mic), higher grade (OR 1.63 for poorly differentiated/undifferentiated vs. well differentiated), and more advanced tumor stage (OR 1.33 and 2.16 for T2 and T3-T4c, respectively, each vs. T1), all *p* < 0.001 (Table 2).

Those whose adjuvant chemotherapy recommendation was discordant with their RS score in the low RS group (i.e., high probability of chemotherapy (>50%) despite low RS) were generally younger or had more advanced disease. Supplemental Table 1 shows the predicted probabilities of adjuvant chemotherapy recommendation among the low RS patients for all observed factor combinations and demonstrates in which patient groups the RS testing is unlikely to have clinical impact due to a high probability of adjuvant chemotherapy recommendation regardless of the low RS.

When looking at patients with a RS of 18–25, the effect of patient age on adjuvant chemotherapy recommendation was similar to that seen in the low risk group. Pathologic nodal stage was also significantly associated with adjuvant chemotherapy recommendation in this group, but the effects were slightly less pronounced than in the low RS group. Higher grade was also significantly associated: OR 2.27 for poorly differentiated and OR 1.48 for moderately differentiated, each vs. well differentiated tumors (each *p* < 0.001). After adjustment for other factors, pathologic tumor stage was not significantly associated with adjuvant chemotherapy recommendation in this subgroup. Among intermediate 18–25 RS patients, N1 and N2 patients were more likely to be recommended chemotherapy compared with N1mi disease (OR 1.38 and 3.67, *p* < 0.001). Whereas in patients with intermediate 26–30 RS, nodal stage did not influence chemotherapy decision (Table 2). Within the RS 26–30 group, only younger age was significantly associated with adjuvant chemotherapy recommendation.

The Charlson–Deyo score did not impact chemotherapy recommendation within any RS category after adjustment for age and tumor factors. Similarly, no significant effect on adjuvant chemotherapy recommendation was observed for ILC histology vs. other histologies.

### Temporal changes

As noted, use of RS testing increased over the time period of this study. Separate multivariable models for each calendar year predicting RS testing and tests for interaction with calendar year showed that progesterone receptor (PR) positivity, which was not significantly associated with RS testing in 2010 (OR 1.08), became increasingly associated with RS testing in later years (OR 1.13 in 2011, OR 1.32 in 2012, OR 1.54 in 2013, *p*-value for time trend in odds ratios < 0.001).

With regard to adjuvant chemotherapy recommendation, the only effect noted to change over time was the odds ratio for pN1 vs. pN1mic, which increased significantly over time in both the low RS (OR 1.2 in 2010 to OR 2.6 in 2013, *p* < 0.001) and intermediate 18–25 RS (OR 0.9 in 2010 to OR 1.5 in 2013, *p* = 0.04) groups.

### RS testing and chemotherapy recommendations in male patients

There were *n* = 1123 males who met the study inclusion criteria. Of these, RS testing was performed in 153 (13.6%), which was significantly lower than the percentage tested in females (20.6%, *p* < 0.001). As with females, males with RS testing were significantly less likely to have adjuvant chemotherapy recommended than those without testing (47.1% vs. 75.5%, *p* < 0.001). The size of the RS testing effect on adjuvant chemotherapy recommendation did not differ between males and females (OR 0.29 vs. 0.24, *p* = 0.28), despite the fact that males were less likely than females to be recommended adjuvant chemotherapy overall (71.6% vs. 74.7%, *p* = 0.02). Among those with a numeric score available, the RS were not significantly different between males and females (median 14.0 (IQR 7.0 to 25.0) vs. median 15.0 (IQR 11.0 to 21.0), respectively, *p* = 0.30). Among males, 60.0% were classified as low RS, 17.6% intermediate 18–25 RS, 11.2% intermediate 26–30 RS, and 11.2% high RS.

## Discussion

Chemotherapy is standardly recommended for ER+, node positive breast cancer based on a meta-analysis of multiple prospective clinical studies demonstrating that poly-chemotherapy significantly reduces breast cancer mortality for ER+, node positive breast cancer.^5^ However, multiple studies have demonstrated that multigene signatures identify a subset of women with node positive, ER+ breast cancer with not only a low risk of distant recurrence but potentially a minimal benefit from adjuvant chemotherapy.^17–19^ While prospective clinical trials^24,25^ are ongoing to evaluate the benefit of utilizing RS to select candidates for adjuvant chemotherapy in node positive breast cancer, multiple studies^17–20,22^ have demonstrated that women with ER+, node positive breast cancer with a low RS (0–18) have a low risk of breast cancer mortality.

Our findings demonstrate that recommendation rate for adjuvant chemotherapy was significantly lower in patients where RS testing was performed. RS results are especially impacting recommendations in patients with low volume nodal disease, while in more advanced nodal disease adjuvant chemotherapy is recommended in the majority of patients regardless of RS. For the intermediate RS group, oncologists appear to be treating 18–25 similar to low RS and 26–30 similar to high RS group. While designing the RxPonder trial,^24,26^ the investigators examined the data of SWOG 8814 and with complex modeling predicted that the chemotherapy benefit is very low in those with RS ≤ 25. Hence it is likely the oncologists’ were using this cutoff of RS in not recommending chemotherapy to the intermediate RS group with scores 18–25.

Our study is a large study of 72,897 patients with ER+/HER2−, node positive breast cancer, looking at the utilization of RS assay in adjuvant chemotherapy decision. Several smaller studies previously have shown similar findings^27–30^ Jasem et al.^31^ using the NCDB, showed that in node negative breast cancer patients, RS assay was ordered in 54% of patients and RS score use was higher in T2, N0(i+) and grade 2+ disease. In our study in node positive patients, RS assay was obtained only in 20.6% of patients. This likely reflects the practice pattern of prescribing chemotherapy to most node positive patients even with estrogen sensitivity. In terms of which patients get tested, a Carolina population based study^32^ from 2008-14 found that in node positive patients, black patients were less likely to undergo RS testing. This was similar to the findings in our study with testing more commonly performed in white patients than black patients in the NCDB data.

How are oncologists using the RS results to tailor adjuvant chemotherapy recommendations? There are data from several small studies to date examining this. In a study from Israel of patients with 1–3 positive lymph nodes, adjuvant chemotherapy was delivered in 70.1% of the 669 control patients, compared to 24.5% in the 282 patients who had RS testing.^30^ The data from our study analyzing the NCDB similarly demonstrated less chemotherapy recommendation in those who had RS testing (50.4%) compared to those without RS testing (81.0%) in node-positive, ER+/HER2− breast cancer.

Studies worldwide tracking physician recommendations for chemotherapy in node positive, ER+/HER2− patients have shown that there is a change in recommendation for chemotherapy before and after RS assay of about 20–50%. The most common change was a switch from chemotherapy and endocrine therapy to endocrine therapy alone.^29,33–37^

Chen et al.^38^ analyzed the NCDB for practice patterns and outcomes in node negative ER+/HER2− breast cancer patients with RS 11–25. They showed that the RS group, age, race, insurance, treatment at a community cancer program, year of diagnosis, grade of tumor, lymphovascular invasion and those having mastectomy and radiation therapy correlated with chemotherapy use. From 2009 to 2013 the percentage of patients receiving chemotherapy decreased (31–18.4%, *p* < 0.001). In the subset of patients with median follow up of 46.4 months, chemotherapy was not associated with improvement in overall survival (*p* = 0.89).

In our study, we found that RS testing was obtained more in patients with lower Charlson–Deyo score, in those with private/managed care insurance, in those treated at academic/research facilities and in older patients with the exception of those ≥ 80 years where use decreased. Testing increased across the time period from 2010–2013. Also, patients with lower tumor stage, lower nodal stage, PR positive, and well-differentiated tumors were more likely to undergo testing.

The distribution of RS in our study was similar to the SEER/Genomic Health linkage study^20^ where 57% had low RS, 36% intermediate RS and 7% with high RS. The distribution of RS was different in the SWOG 8814 study,^19^ where 39.8% had low RS, 28.1% intermediate RS and 32.2% high RS likely reflecting the selection of patients enrolling in a clinical trial and the fact RS testing was done only in a subset of patients. Also, in the SWOG 8814 study 20.4% were PR negative whereas in our study only 7.3% were PR negative in those who had RS testing reflecting slightly different subsets of patients in the two studies. In the SWOG study, 11.7% of the blocks tested for RS were HER2-positive by the RT-PCR assay, which could have contributed to this difference in distribution of RS as well.

Histology was not significantly associated with chemotherapy recommendation in any RS group after adjustment for other factors. Younger age was associated with increased odds of chemotherapy recommendation in low and intermediate RS groups. Also, in low RS and RS 18–25 patients those with higher grade of tumor, N1or N2 vs. N1mi were more likely to be recommended chemotherapy. Interestingly, pathologic nodal stage was not associated with chemotherapy recommendation in the 26–30 RS group.

The NCDB data demonstrates that clinicians are treating patients with N2/N3 disease with chemotherapy regardless of RS and other clinical and pathologic features. This calls into question the benefit of ordering RS assay in these patients if physicians/patients are reluctant to adjust treatment recommendations based on the test results. Results from the ongoing prospective trials are required to evaluate whether RS should be used to guide adjuvant chemotherapy recommendations in patients with higher volume nodal involvement.

Patients with an intermediate RS remain the most challenging from a clinical decision making standpoint as the least data is available in this group. Interestingly, analyzing the intermediate RS group as low intermediate (18–25) and high intermediate (26–30) showed that patients with RS 26–30 were treated like those high RS (>30) with the vast majority being recommended adjuvant chemotherapy. On the other hand, recommendations for patients with tumors with RS 18–25 were treated similar to those with low RS (<18). Given the role for extended adjuvant hormonal therapy, with further 15–40% reductions in recurrence for women receiving either tamoxifen or AI’s in years 5–10,^2–4^ the absolute benefit of chemotherapy regimens in this subgroup may be small.

The ongoing prospective randomized study S1007 or RxPONDER (Rx for Positive Node, ER+ Breast Cancer)^24^ which randomizes patients with ER+/HER2− negative breast cancer with 1–3 positive lymph nodes, with RS ≤ 25 to either endocrine therapy alone or chemotherapy followed by endocrine therapy will provide prospective data on the most appropriate systemic therapy recommendations for this group. The OPTIMA (Optimal Personalised Treatment of early Breast Cancer using Multiparameter Analysis) trial^25^ opened in the UK in 2012 wherein patients with ER+/HER2−, pN1-2 or pN0 *T* > 30 mm are being randomized to standard therapy (chemotherapy and endocrine therapy) vs. test-directed therapy (chemotherapy for high risk patients only) with a goal to find the most cost-effective gene assay (RS compared to others) to define high risk women that benefit from chemotherapy for ER+/HER2− breast cancer.

Our study has several limitations. Some degree of missing or inconsistent data is inevitable in a database as large as the NCDB, which includes reports from more than 1500 facilities. RS assay is a relatively new variable, only added to NCDB as a breast site-specific factor in 2010; thus there is the potential for missing data or coding errors. Further, among those tested, the absolute RS value was not available for all patients. One weakness of our study is that we did not have information regarding quantification of ER which may have influenced decision to obtain RS testing. Further, since outcome data such as local-regional and distant recurrence is not available from NCDB, and the follow-up was short, the impact of RS and the choice regarding adjuvant chemotherapy on outcomes was not assessed. Finally, we were unable to observe the decision making of medical oncologists or patients, only practice patterns.

Our study evaluating the NCDB data demonstrates that oncologists are already obtaining RS assay in approximately 1 in 5 node positive, ER+/HER2− negative breast cancer patients and are using the RS results to tailor treatment recommendations, in parallel to ongoing prospective randomized trials. There was a large impact in decreasing chemotherapy recommendation in those who had RS assay compared to those who did not have the assay. While we have ample evidence of prognostic benefit of RS in N+ patients, it remains to be seen if ongoing prospective studies of multi-gene signatures in node positive breast cancer demonstrate a predictive benefit of RS testing for use of adjuvant chemotherapy and allow us to reliably spare low risk individuals the burden of chemotherapy where no benefit exists.

## Methods

We analyzed female patients with pathologic stage T1-T4c, N1mi-N3, ER+/HER2− breast cancer diagnosed between 2010 and 2013 and who were included in the NCDB participant user file (PUF). Our Institutional Review Board has deemed analysis of the NCDB PUFs exempt from review. The NCDB is a nationwide cancer database sponsored by the Commission on Cancer of the American College of Surgeons and the American Cancer Society. NCDB cases represent approximately 70% of newly diagnosed cancer cases nationwide.^39^ It contains over 30 million records of individual cancer cases collected by more than 1500 Commission on Cancer (CoC) approved facilities across the United States (US). NCDB data reporting is tracked, audited, and must meet quality standards in order for centers to maintain their CoC center designation.^39^

Patients with invasive breast carcinoma were identified using International Classification of Diseases for Oncology, 3rd Edition (ICD-O-3) topography (C50.0-50.9) and histology (8000–8576, 8940–8950, 8980–8981) codes. Only patients receiving treatment at the reporting facility were included. Patients diagnosed with cancer at more than one primary site, those that did not receive treatment at the reporting facility, those missing data on pathologic staging, cases of inflammatory or phyllodes breast cancer, and patients who received neoadjuvant treatment (chemotherapy, hormone therapy, or radiation) were excluded. Comorbidities were assessed using the method outlined by Charlson^40^ and Deyo,^41^ and was categorized by NCDB as 0, 1, or ≥2 comorbidities. AJCC pathologic staging was assessed according to the 7th edition AJCC staging manual.

The NCDB started collection of RS data in 2010. Patients were coded according to whether adjuvant chemotherapy was recommended as part of the first course of therapy (which is a specific field collected in the NCDB). Chemotherapy recommendation rather than chemotherapy received was used for the primary analysis since the goal of this study was to assess impact on provider treatment decisions.

The RS assays were classified as low (<18), intermediate (18–30), and high (>30). The intermediate group was further divided into low intermediate and high intermediate (those with scores ranging from 18–25 and those with scores 26–30, respectively) based on cutoffs used in ongoing clinical trial in N+ breast cancer.^24,26^

Additionally, a separate analysis was performed evaluating RS testing and impact on chemotherapy recommendation in male patients in NCDB.

### Statistical analysis

Variables were summarized descriptively using frequencies and proportions for categorical variables and median with IQR for continuous variables. Analyses were performed comparing patients with and without RS testing and subsequently focused on those patients with RS testing and a numeric RS available to evaluate for impact of test results on recommendation for chemotherapy. Analyses included Cochrane-Armitage tests for linear trend over time and multivariable logistic regression assessing factors influencing RS testing. Similarly, multivariable logistic regression was used to assess factors influencing adjuvant chemotherapy recommendations within RS strata. As the number of variables available in NCDB was relatively small and the available sample size was large, all clinically relevant variables were included in models without use of variable selection algorithms. Results were reported with odds ratios (ORs) and 95% confidence intervals. Interaction terms were used to assess whether any factors had differential effects across calendar years. Predicted probabilities of adjuvant chemotherapy recommendation were derived as a function of the coefficients from the multivariable logistic regression model. Wilcoxon rank-sum tests for continuous variables or chi-square tests for dichotomous variables were used for basic two-sample comparisons. The Breslow–Day test for homogeneity of odds ratios was used to compare the effect of RS testing on adjuvant chemotherapy recommendation between males and females. Analysis was performed using SAS (Version 9.4, SAS Institute Inc., Cary, NC). All tests were two-sided and *p*-values < 0.05 were considered significant.

Given our study design was a retrospective cohort study of observational data from a large national database, we did not choose a specific sample necessary to target a specific effect size but rather utilized all patients who met our criteria in the NCDB. However, as ten events per variable is generally considered an adequate sample size for multivariable logistic regression,^42^ this criterion was exceeded for all primary analyses.

### Data availability statement

NCDB PUFs are only available through an application process to investigators associated with CoC-accredited cancer programs.^43^ The Request for Applications occurs on a semi-annual basis, and PUFs are referred to by their most recent year of diagnosis included in the data file.

## Electronic supplementary material

Supplemental Table 1
