# Supplementary material for: Use of 21-gene recurrence score assay to individualize adjuvant chemotherapy recommendations in ER+/HER2− node positive breast cancer—A National Cancer Database study
Source: NPJ Breast Cancer. 2017 Oct 19;3:41. doi: 10.1038/s41523-017-0044-4 (PMC5648884; doi:10.1038/s41523-017-0044-4)
Supplement: Supplementary file 1 — Supplemental Table 1 [file 41523_2017_44_MOESM1_ESM.docx]

Supplemental Table 1: Predicted Probabilities of Adjuvant Chemotherapy Recommendation Among Low Risk RS Patients

| **Age Group** | **Pathologic N Stage** | **Pathologic T Stage** | **Grade** | **Charlson-Deyo Score** | **ILC Histology** | **Predicted Probability (%) of Adjuvant Chemotherapy** |
| --- | --- | --- | --- | --- | --- | --- |
| Under 40 | N1mi | T0/T1 | Well differentiated | 0 | Other than ILC | 46 |
| Under 40 | N1mi | T0/T1 | Well differentiated | 1 | Other than ILC | 45 |
| Under 40 | N1mi | T0/T1 | Moderately differentiated | 0 | Other than ILC | 50 |
| Under 40 | N1mi | T0/T1 | Moderately differentiated | 1 | Other than ILC | 49 |
| Under 40 | N1mi | T0/T1 | Moderately differentiated | 2+ | Other than ILC | 51 |
| Under 40 | N1mi | T0/T1 | Poorly differentiated / Undifferentiated | 0 | Other than ILC | 59 |
| Under 40 | N1mi | T0/T1 | Cell type not determined | 0 | Other than ILC | 48 |
| Under 40 | N1mi | T2 | Well differentiated | 0 | Other than ILC | 54 |
| Under 40 | N1mi | T2 | Well differentiated | 1 | Other than ILC | 52 |
| Under 40 | N1mi | T2 | Moderately differentiated | 0 | Other than ILC | 58 |
| Under 40 | N1mi | T2 | Moderately differentiated | 2+ | Other than ILC | 58 |
| Under 40 | N1mi | T2 | Poorly differentiated / Undifferentiated | 0 | Other than ILC | 65 |
| Under 40 | N1mi | T2 | Cell type not determined | 0 | Other than ILC | 55 |
| Under 40 | N1 | T0/T1 | Well differentiated | 0 | Other than ILC | 66 |
| Under 40 | N1 | T0/T1 | Moderately differentiated | 0 | ILC | 68 |
| Under 40 | N1 | T0/T1 | Moderately differentiated | 0 | Other than ILC | 70 |
| Under 40 | N1 | T0/T1 | Moderately differentiated | 1 | Other than ILC | 69 |
| Under 40 | N1 | T0/T1 | Poorly differentiated / Undifferentiated | 0 | Other than ILC | 76 |
| Under 40 | N1 | T0/T1 | Cell type not determined | 0 | Other than ILC | 68 |
| Under 40 | N1 | T2 | Well differentiated | 0 | Other than ILC | 72 |
| Under 40 | N1 | T2 | Moderately differentiated | 0 | Other than ILC | 75 |
| Under 40 | N1 | T2 | Moderately differentiated | 1 | Other than ILC | 74 |
| Under 40 | N1 | T2 | Poorly differentiated / Undifferentiated | 0 | Other than ILC | 81 |
| Under 40 | N1 | T2 | Poorly differentiated / Undifferentiated | 1 | ILC | 79 |
| Under 40 | N1 | T2 | Poorly differentiated / Undifferentiated | 1 | Other than ILC | 80 |
| Under 40 | N1 | T2 | Cell type not determined | 0 | ILC | 72 |
| Under 40 | N1 | T3/T4 | Moderately differentiated | 0 | Other than ILC | 83 |
| Under 40 | N1 | T3/T4 | Poorly differentiated / Undifferentiated | 0 | Other than ILC | 87 |
| Under 40 | N2+ | T0/T1 | Moderately differentiated | 0 | Other than ILC | 90 |
| Under 40 | N2+ | T2 | Moderately differentiated | 0 | Other than ILC | 92 |
| Under 40 | N2+ | T2 | Poorly differentiated / Undifferentiated | 0 | Other than ILC | 94 |
| Under 40 | N2+ | T2 | Poorly differentiated / Undifferentiated | 1 | Other than ILC | 94 |
| Under 40 | N2+ | T3/T4 | Cell type not determined | 0 | Other than ILC | 95 |
| 40-49 | N1mi | T0/T1 | Well differentiated | 0 | ILC | 30 |
| 40-49 | N1mi | T0/T1 | Well differentiated | 0 | Other than ILC | 32 |
| 40-49 | N1mi | T0/T1 | Well differentiated | 1 | Other than ILC | 31 |
| 40-49 | N1mi | T0/T1 | Well differentiated | 2+ | Other than ILC | 33 |
| 40-49 | N1mi | T0/T1 | Moderately differentiated | 0 | ILC | 34 |
| 40-49 | N1mi | T0/T1 | Moderately differentiated | 0 | Other than ILC | 36 |
| 40-49 | N1mi | T0/T1 | Moderately differentiated | 1 | ILC | 33 |
| 40-49 | N1mi | T0/T1 | Moderately differentiated | 1 | Other than ILC | 35 |
| 40-49 | N1mi | T0/T1 | Moderately differentiated | 2+ | Other than ILC | 36 |
| 40-49 | N1mi | T0/T1 | Poorly differentiated / Undifferentiated | 0 | Other than ILC | 44 |
| 40-49 | N1mi | T0/T1 | Poorly differentiated / Undifferentiated | 2+ | Other than ILC | 44 |
| 40-49 | N1mi | T0/T1 | Cell type not determined | 0 | ILC | 32 |
| 40-49 | N1mi | T0/T1 | Cell type not determined | 0 | Other than ILC | 34 |
| 40-49 | N1mi | T0/T1 | Cell type not determined | 2+ | Other than ILC | 34 |
| 40-49 | N1mi | T2 | Well differentiated | 0 | ILC | 37 |
| 40-49 | N1mi | T2 | Well differentiated | 0 | Other than ILC | 39 |
| 40-49 | N1mi | T2 | Well differentiated | 1 | ILC | 36 |
| 40-49 | N1mi | T2 | Well differentiated | 1 | Other than ILC | 37 |
| 40-49 | N1mi | T2 | Moderately differentiated | 0 | ILC | 41 |
| 40-49 | N1mi | T2 | Moderately differentiated | 0 | Other than ILC | 43 |
| 40-49 | N1mi | T2 | Moderately differentiated | 1 | Other than ILC | 41 |
| 40-49 | N1mi | T2 | Poorly differentiated / Undifferentiated | 0 | Other than ILC | 51 |
| 40-49 | N1mi | T2 | Poorly differentiated / Undifferentiated | 1 | Other than ILC | 49 |
| 40-49 | N1mi | T2 | Cell type not determined | 0 | ILC | 38 |
| 40-49 | N1mi | T2 | Cell type not determined | 0 | Other than ILC | 40 |
| 40-49 | N1mi | T2 | Cell type not determined | 2+ | Other than ILC | 41 |
| 40-49 | N1mi | T3/T4 | Well differentiated | 0 | ILC | 49 |
| 40-49 | N1mi | T3/T4 | Moderately differentiated | 0 | ILC | 53 |
| 40-49 | N1mi | T3/T4 | Moderately differentiated | 0 | Other than ILC | 55 |
| 40-49 | N1mi | T3/T4 | Poorly differentiated / Undifferentiated | 0 | Other than ILC | 62 |
| 40-49 | N1 | T0/T1 | Well differentiated | 0 | ILC | 50 |
| 40-49 | N1 | T0/T1 | Well differentiated | 0 | Other than ILC | 52 |
| 40-49 | N1 | T0/T1 | Well differentiated | 1 | ILC | 48 |
| 40-49 | N1 | T0/T1 | Well differentiated | 1 | Other than ILC | 50 |
| 40-49 | N1 | T0/T1 | Well differentiated | 2+ | ILC | 50 |
| 40-49 | N1 | T0/T1 | Well differentiated | 2+ | Other than ILC | 52 |
| 40-49 | N1 | T0/T1 | Moderately differentiated | 0 | ILC | 54 |
| 40-49 | N1 | T0/T1 | Moderately differentiated | 0 | Other than ILC | 56 |
| 40-49 | N1 | T0/T1 | Moderately differentiated | 1 | ILC | 52 |
| 40-49 | N1 | T0/T1 | Moderately differentiated | 1 | Other than ILC | 54 |
| 40-49 | N1 | T0/T1 | Moderately differentiated | 2+ | Other than ILC | 56 |
| 40-49 | N1 | T0/T1 | Poorly differentiated / Undifferentiated | 0 | Other than ILC | 63 |
| 40-49 | N1 | T0/T1 | Poorly differentiated / Undifferentiated | 1 | ILC | 60 |
| 40-49 | N1 | T0/T1 | Poorly differentiated / Undifferentiated | 1 | Other than ILC | 62 |
| 40-49 | N1 | T0/T1 | Cell type not determined | 0 | ILC | 51 |
| 40-49 | N1 | T0/T1 | Cell type not determined | 0 | Other than ILC | 53 |
| 40-49 | N1 | T0/T1 | Cell type not determined | 1 | Other than ILC | 52 |
| 40-49 | N1 | T2 | Well differentiated | 0 | ILC | 57 |
| 40-49 | N1 | T2 | Well differentiated | 0 | Other than ILC | 59 |
| 40-49 | N1 | T2 | Well differentiated | 2+ | Other than ILC | 59 |
| 40-49 | N1 | T2 | Moderately differentiated | 0 | ILC | 61 |
| 40-49 | N1 | T2 | Moderately differentiated | 0 | Other than ILC | 63 |
| 40-49 | N1 | T2 | Moderately differentiated | 1 | ILC | 59 |
| 40-49 | N1 | T2 | Moderately differentiated | 1 | Other than ILC | 61 |
| 40-49 | N1 | T2 | Moderately differentiated | 2+ | Other than ILC | 63 |
| 40-49 | N1 | T2 | Poorly differentiated / Undifferentiated | 0 | ILC | 68 |
| 40-49 | N1 | T2 | Poorly differentiated / Undifferentiated | 0 | Other than ILC | 70 |
| 40-49 | N1 | T2 | Poorly differentiated / Undifferentiated | 1 | ILC | 67 |
| 40-49 | N1 | T2 | Poorly differentiated / Undifferentiated | 1 | Other than ILC | 69 |
| 40-49 | N1 | T2 | Poorly differentiated / Undifferentiated | 2+ | Other than ILC | 70 |
| 40-49 | N1 | T2 | Cell type not determined | 0 | ILC | 58 |
| 40-49 | N1 | T2 | Cell type not determined | 0 | Other than ILC | 60 |
| 40-49 | N1 | T2 | Cell type not determined | 1 | ILC | 57 |
| 40-49 | N1 | T2 | Cell type not determined | 1 | Other than ILC | 59 |
| 40-49 | N1 | T3/T4 | Well differentiated | 0 | ILC | 68 |
| 40-49 | N1 | T3/T4 | Well differentiated | 0 | Other than ILC | 70 |
| 40-49 | N1 | T3/T4 | Moderately differentiated | 0 | ILC | 71 |
| 40-49 | N1 | T3/T4 | Moderately differentiated | 0 | Other than ILC | 73 |
| 40-49 | N1 | T3/T4 | Moderately differentiated | 1 | Other than ILC | 72 |
| 40-49 | N1 | T3/T4 | Moderately differentiated | 2+ | ILC | 72 |
| 40-49 | N1 | T3/T4 | Poorly differentiated / Undifferentiated | 0 | ILC | 78 |
| 40-49 | N1 | T3/T4 | Poorly differentiated / Undifferentiated | 0 | Other than ILC | 79 |
| 40-49 | N1 | T3/T4 | Cell type not determined | 0 | Other than ILC | 71 |
| 40-49 | N2+ | T0/T1 | Well differentiated | 0 | ILC | 80 |
| 40-49 | N2+ | T0/T1 | Well differentiated | 0 | Other than ILC | 81 |
| 40-49 | N2+ | T0/T1 | Moderately differentiated | 0 | ILC | 82 |
| 40-49 | N2+ | T0/T1 | Moderately differentiated | 0 | Other than ILC | 83 |
| 40-49 | N2+ | T0/T1 | Moderately differentiated | 1 | Other than ILC | 83 |
| 40-49 | N2+ | T0/T1 | Poorly differentiated / Undifferentiated | 0 | ILC | 86 |
| 40-49 | N2+ | T2 | Well differentiated | 0 | ILC | 84 |
| 40-49 | N2+ | T2 | Well differentiated | 0 | Other than ILC | 85 |
| 40-49 | N2+ | T2 | Well differentiated | 1 | Other than ILC | 84 |
| 40-49 | N2+ | T2 | Moderately differentiated | 0 | ILC | 86 |
| 40-49 | N2+ | T2 | Moderately differentiated | 0 | Other than ILC | 87 |
| 40-49 | N2+ | T2 | Moderately differentiated | 1 | Other than ILC | 86 |
| 40-49 | N2+ | T2 | Poorly differentiated / Undifferentiated | 2+ | Other than ILC | 90 |
| 40-49 | N2+ | T2 | Cell type not determined | 0 | Other than ILC | 86 |
| 40-49 | N2+ | T3/T4 | Well differentiated | 0 | ILC | 89 |
| 40-49 | N2+ | T3/T4 | Moderately differentiated | 0 | Other than ILC | 91 |
| 40-49 | N2+ | T3/T4 | Moderately differentiated | 1 | Other than ILC | 91 |
| 40-49 | N2+ | T3/T4 | Cell type not determined | 0 | Other than ILC | 91 |
| 50-59 | N1mi | T0/T1 | Well differentiated | 0 | ILC | 20 |
| 50-59 | N1mi | T0/T1 | Well differentiated | 0 | Other than ILC | 22 |
| 50-59 | N1mi | T0/T1 | Well differentiated | 1 | Other than ILC | 21 |
| 50-59 | N1mi | T0/T1 | Moderately differentiated | 0 | ILC | 23 |
| 50-59 | N1mi | T0/T1 | Moderately differentiated | 0 | Other than ILC | 25 |
| 50-59 | N1mi | T0/T1 | Moderately differentiated | 1 | ILC | 22 |
| 50-59 | N1mi | T0/T1 | Moderately differentiated | 1 | Other than ILC | 24 |
| 50-59 | N1mi | T0/T1 | Moderately differentiated | 2+ | ILC | 24 |
| 50-59 | N1mi | T0/T1 | Moderately differentiated | 2+ | Other than ILC | 25 |
| 50-59 | N1mi | T0/T1 | Poorly differentiated / Undifferentiated | 0 | Other than ILC | 31 |
| 50-59 | N1mi | T0/T1 | Poorly differentiated / Undifferentiated | 1 | Other than ILC | 30 |
| 50-59 | N1mi | T0/T1 | Cell type not determined | 0 | ILC | 21 |
| 50-59 | N1mi | T0/T1 | Cell type not determined | 0 | Other than ILC | 23 |
| 50-59 | N1mi | T0/T1 | Cell type not determined | 1 | Other than ILC | 22 |
| 50-59 | N1mi | T2 | Well differentiated | 0 | ILC | 25 |
| 50-59 | N1mi | T2 | Well differentiated | 0 | Other than ILC | 27 |
| 50-59 | N1mi | T2 | Well differentiated | 1 | Other than ILC | 26 |
| 50-59 | N1mi | T2 | Well differentiated | 2+ | Other than ILC | 27 |
| 50-59 | N1mi | T2 | Moderately differentiated | 0 | ILC | 29 |
| 50-59 | N1mi | T2 | Moderately differentiated | 0 | Other than ILC | 30 |
| 50-59 | N1mi | T2 | Moderately differentiated | 1 | ILC | 27 |
| 50-59 | N1mi | T2 | Moderately differentiated | 1 | Other than ILC | 29 |
| 50-59 | N1mi | T2 | Moderately differentiated | 2+ | ILC | 29 |
| 50-59 | N1mi | T2 | Moderately differentiated | 2+ | Other than ILC | 31 |
| 50-59 | N1mi | T2 | Poorly differentiated / Undifferentiated | 0 | ILC | 36 |
| 50-59 | N1mi | T2 | Poorly differentiated / Undifferentiated | 0 | Other than ILC | 37 |
| 50-59 | N1mi | T2 | Poorly differentiated / Undifferentiated | 1 | Other than ILC | 36 |
| 50-59 | N1mi | T2 | Poorly differentiated / Undifferentiated | 2+ | Other than ILC | 38 |
| 50-59 | N1mi | T2 | Cell type not determined | 0 | ILC | 27 |
| 50-59 | N1mi | T2 | Cell type not determined | 0 | Other than ILC | 28 |
| 50-59 | N1mi | T2 | Cell type not determined | 1 | Other than ILC | 27 |
| 50-59 | N1mi | T3/T4 | Well differentiated | 0 | ILC | 36 |
| 50-59 | N1mi | T3/T4 | Well differentiated | 0 | Other than ILC | 37 |
| 50-59 | N1mi | T3/T4 | Moderately differentiated | 0 | ILC | 39 |
| 50-59 | N1mi | T3/T4 | Moderately differentiated | 0 | Other than ILC | 41 |
| 50-59 | N1mi | T3/T4 | Moderately differentiated | 1 | ILC | 38 |
| 50-59 | N1mi | T3/T4 | Poorly differentiated / Undifferentiated | 0 | Other than ILC | 49 |
| 50-59 | N1 | T0/T1 | Well differentiated | 0 | ILC | 37 |
| 50-59 | N1 | T0/T1 | Well differentiated | 0 | Other than ILC | 38 |
| 50-59 | N1 | T0/T1 | Well differentiated | 1 | ILC | 35 |
| 50-59 | N1 | T0/T1 | Well differentiated | 1 | Other than ILC | 37 |
| 50-59 | N1 | T0/T1 | Well differentiated | 2+ | Other than ILC | 39 |
| 50-59 | N1 | T0/T1 | Moderately differentiated | 0 | ILC | 40 |
| 50-59 | N1 | T0/T1 | Moderately differentiated | 0 | Other than ILC | 42 |
| 50-59 | N1 | T0/T1 | Moderately differentiated | 1 | ILC | 39 |
| 50-59 | N1 | T0/T1 | Moderately differentiated | 1 | Other than ILC | 41 |
| 50-59 | N1 | T0/T1 | Moderately differentiated | 2+ | ILC | 41 |
| 50-59 | N1 | T0/T1 | Moderately differentiated | 2+ | Other than ILC | 43 |
| 50-59 | N1 | T0/T1 | Poorly differentiated / Undifferentiated | 0 | ILC | 48 |
| 50-59 | N1 | T0/T1 | Poorly differentiated / Undifferentiated | 0 | Other than ILC | 50 |
| 50-59 | N1 | T0/T1 | Poorly differentiated / Undifferentiated | 1 | Other than ILC | 49 |
| 50-59 | N1 | T0/T1 | Poorly differentiated / Undifferentiated | 2+ | Other than ILC | 51 |
| 50-59 | N1 | T0/T1 | Cell type not determined | 0 | ILC | 38 |
| 50-59 | N1 | T0/T1 | Cell type not determined | 0 | Other than ILC | 40 |
| 50-59 | N1 | T0/T1 | Cell type not determined | 1 | Other than ILC | 39 |
| 50-59 | N1 | T0/T1 | Cell type not determined | 2+ | Other than ILC | 41 |
| 50-59 | N1 | T2 | Well differentiated | 0 | ILC | 43 |
| 50-59 | N1 | T2 | Well differentiated | 0 | Other than ILC | 45 |
| 50-59 | N1 | T2 | Well differentiated | 1 | ILC | 42 |
| 50-59 | N1 | T2 | Well differentiated | 1 | Other than ILC | 44 |
| 50-59 | N1 | T2 | Well differentiated | 2+ | Other than ILC | 46 |
| 50-59 | N1 | T2 | Moderately differentiated | 0 | ILC | 47 |
| 50-59 | N1 | T2 | Moderately differentiated | 0 | Other than ILC | 49 |
| 50-59 | N1 | T2 | Moderately differentiated | 1 | ILC | 46 |
| 50-59 | N1 | T2 | Moderately differentiated | 1 | Other than ILC | 48 |
| 50-59 | N1 | T2 | Moderately differentiated | 2+ | ILC | 48 |
| 50-59 | N1 | T2 | Moderately differentiated | 2+ | Other than ILC | 50 |
| 50-59 | N1 | T2 | Poorly differentiated / Undifferentiated | 0 | ILC | 55 |
| 50-59 | N1 | T2 | Poorly differentiated / Undifferentiated | 0 | Other than ILC | 57 |
| 50-59 | N1 | T2 | Poorly differentiated / Undifferentiated | 1 | ILC | 54 |
| 50-59 | N1 | T2 | Poorly differentiated / Undifferentiated | 1 | Other than ILC | 56 |
| 50-59 | N1 | T2 | Poorly differentiated / Undifferentiated | 2+ | Other than ILC | 58 |
| 50-59 | N1 | T2 | Cell type not determined | 0 | ILC | 45 |
| 50-59 | N1 | T2 | Cell type not determined | 0 | Other than ILC | 47 |
| 50-59 | N1 | T2 | Cell type not determined | 1 | Other than ILC | 46 |
| 50-59 | N1 | T2 | Cell type not determined | 2+ | Other than ILC | 48 |
| 50-59 | N1 | T3/T4 | Well differentiated | 0 | ILC | 55 |
| 50-59 | N1 | T3/T4 | Well differentiated | 0 | Other than ILC | 57 |
| 50-59 | N1 | T3/T4 | Moderately differentiated | 0 | ILC | 59 |
| 50-59 | N1 | T3/T4 | Moderately differentiated | 0 | Other than ILC | 61 |
| 50-59 | N1 | T3/T4 | Moderately differentiated | 1 | ILC | 58 |
| 50-59 | N1 | T3/T4 | Moderately differentiated | 1 | Other than ILC | 60 |
| 50-59 | N1 | T3/T4 | Poorly differentiated / Undifferentiated | 0 | Other than ILC | 69 |
| 50-59 | N1 | T3/T4 | Cell type not determined | 0 | ILC | 57 |
| 50-59 | N2+ | T0/T1 | Well differentiated | 0 | Other than ILC | 71 |
| 50-59 | N2+ | T0/T1 | Well differentiated | 1 | Other than ILC | 70 |
| 50-59 | N2+ | T0/T1 | Moderately differentiated | 0 | ILC | 73 |
| 50-59 | N2+ | T0/T1 | Moderately differentiated | 0 | Other than ILC | 74 |
| 50-59 | N2+ | T0/T1 | Moderately differentiated | 1 | Other than ILC | 73 |
| 50-59 | N2+ | T0/T1 | Poorly differentiated / Undifferentiated | 0 | Other than ILC | 80 |
| 50-59 | N2+ | T2 | Well differentiated | 0 | ILC | 75 |
| 50-59 | N2+ | T2 | Well differentiated | 0 | Other than ILC | 77 |
| 50-59 | N2+ | T2 | Well differentiated | 2+ | Other than ILC | 77 |
| 50-59 | N2+ | T2 | Moderately differentiated | 0 | ILC | 78 |
| 50-59 | N2+ | T2 | Moderately differentiated | 0 | Other than ILC | 79 |
| 50-59 | N2+ | T2 | Moderately differentiated | 1 | Other than ILC | 79 |
| 50-59 | N2+ | T2 | Poorly differentiated / Undifferentiated | 1 | Other than ILC | 84 |
| 50-59 | N2+ | T3/T4 | Well differentiated | 0 | ILC | 83 |
| 50-59 | N2+ | T3/T4 | Moderately differentiated | 0 | ILC | 85 |
| 50-59 | N2+ | T3/T4 | Moderately differentiated | 0 | Other than ILC | 86 |
| 50-59 | N2+ | T3/T4 | Moderately differentiated | 1 | ILC | 85 |
| 50-59 | N2+ | T3/T4 | Poorly differentiated / Undifferentiated | 0 | Other than ILC | 90 |
| 60-69 | N1mi | T0/T1 | Well differentiated | 0 | ILC | 15 |
| 60-69 | N1mi | T0/T1 | Well differentiated | 0 | Other than ILC | 16 |
| 60-69 | N1mi | T0/T1 | Well differentiated | 1 | Other than ILC | 15 |
| 60-69 | N1mi | T0/T1 | Well differentiated | 2+ | Other than ILC | 16 |
| 60-69 | N1mi | T0/T1 | Moderately differentiated | 0 | ILC | 17 |
| 60-69 | N1mi | T0/T1 | Moderately differentiated | 0 | Other than ILC | 18 |
| 60-69 | N1mi | T0/T1 | Moderately differentiated | 1 | ILC | 16 |
| 60-69 | N1mi | T0/T1 | Moderately differentiated | 1 | Other than ILC | 17 |
| 60-69 | N1mi | T0/T1 | Moderately differentiated | 2+ | ILC | 17 |
| 60-69 | N1mi | T0/T1 | Moderately differentiated | 2+ | Other than ILC | 19 |
| 60-69 | N1mi | T0/T1 | Poorly differentiated / Undifferentiated | 0 | ILC | 22 |
| 60-69 | N1mi | T0/T1 | Poorly differentiated / Undifferentiated | 0 | Other than ILC | 24 |
| 60-69 | N1mi | T0/T1 | Poorly differentiated / Undifferentiated | 1 | Other than ILC | 23 |
| 60-69 | N1mi | T0/T1 | Cell type not determined | 0 | ILC | 16 |
| 60-69 | N1mi | T0/T1 | Cell type not determined | 0 | Other than ILC | 17 |
| 60-69 | N1mi | T0/T1 | Cell type not determined | 1 | ILC | 15 |
| 60-69 | N1mi | T0/T1 | Cell type not determined | 1 | Other than ILC | 16 |
| 60-69 | N1mi | T0/T1 | Cell type not determined | 2+ | Other than ILC | 17 |
| 60-69 | N1mi | T2 | Well differentiated | 0 | ILC | 19 |
| 60-69 | N1mi | T2 | Well differentiated | 0 | Other than ILC | 20 |
| 60-69 | N1mi | T2 | Well differentiated | 1 | ILC | 18 |
| 60-69 | N1mi | T2 | Well differentiated | 1 | Other than ILC | 19 |
| 60-69 | N1mi | T2 | Well differentiated | 2+ | ILC | 19 |
| 60-69 | N1mi | T2 | Well differentiated | 2+ | Other than ILC | 21 |
| 60-69 | N1mi | T2 | Moderately differentiated | 0 | ILC | 21 |
| 60-69 | N1mi | T2 | Moderately differentiated | 0 | Other than ILC | 23 |
| 60-69 | N1mi | T2 | Moderately differentiated | 1 | ILC | 21 |
| 60-69 | N1mi | T2 | Moderately differentiated | 1 | Other than ILC | 22 |
| 60-69 | N1mi | T2 | Moderately differentiated | 2+ | ILC | 22 |
| 60-69 | N1mi | T2 | Moderately differentiated | 2+ | Other than ILC | 23 |
| 60-69 | N1mi | T2 | Poorly differentiated / Undifferentiated | 0 | ILC | 27 |
| 60-69 | N1mi | T2 | Poorly differentiated / Undifferentiated | 0 | Other than ILC | 29 |
| 60-69 | N1mi | T2 | Poorly differentiated / Undifferentiated | 1 | ILC | 26 |
| 60-69 | N1mi | T2 | Poorly differentiated / Undifferentiated | 1 | Other than ILC | 28 |
| 60-69 | N1mi | T2 | Poorly differentiated / Undifferentiated | 2+ | Other than ILC | 30 |
| 60-69 | N1mi | T2 | Cell type not determined | 0 | ILC | 20 |
| 60-69 | N1mi | T2 | Cell type not determined | 0 | Other than ILC | 21 |
| 60-69 | N1mi | T2 | Cell type not determined | 1 | ILC | 19 |
| 60-69 | N1mi | T2 | Cell type not determined | 2+ | Other than ILC | 22 |
| 60-69 | N1mi | T3/T4 | Well differentiated | 0 | ILC | 27 |
| 60-69 | N1mi | T3/T4 | Well differentiated | 0 | Other than ILC | 29 |
| 60-69 | N1mi | T3/T4 | Well differentiated | 1 | Other than ILC | 28 |
| 60-69 | N1mi | T3/T4 | Moderately differentiated | 0 | Other than ILC | 32 |
| 60-69 | N1mi | T3/T4 | Moderately differentiated | 1 | ILC | 30 |
| 60-69 | N1 | T0/T1 | Well differentiated | 0 | ILC | 28 |
| 60-69 | N1 | T0/T1 | Well differentiated | 0 | Other than ILC | 30 |
| 60-69 | N1 | T0/T1 | Well differentiated | 1 | ILC | 27 |
| 60-69 | N1 | T0/T1 | Well differentiated | 1 | Other than ILC | 29 |
| 60-69 | N1 | T0/T1 | Well differentiated | 2+ | ILC | 29 |
| 60-69 | N1 | T0/T1 | Well differentiated | 2+ | Other than ILC | 30 |
| 60-69 | N1 | T0/T1 | Moderately differentiated | 0 | ILC | 32 |
| 60-69 | N1 | T0/T1 | Moderately differentiated | 0 | Other than ILC | 33 |
| 60-69 | N1 | T0/T1 | Moderately differentiated | 1 | ILC | 31 |
| 60-69 | N1 | T0/T1 | Moderately differentiated | 1 | Other than ILC | 32 |
| 60-69 | N1 | T0/T1 | Moderately differentiated | 2+ | ILC | 32 |
| 60-69 | N1 | T0/T1 | Moderately differentiated | 2+ | Other than ILC | 34 |
| 60-69 | N1 | T0/T1 | Poorly differentiated / Undifferentiated | 0 | ILC | 39 |
| 60-69 | N1 | T0/T1 | Poorly differentiated / Undifferentiated | 0 | Other than ILC | 41 |
| 60-69 | N1 | T0/T1 | Poorly differentiated / Undifferentiated | 1 | Other than ILC | 40 |
| 60-69 | N1 | T0/T1 | Poorly differentiated / Undifferentiated | 2+ | Other than ILC | 42 |
| 60-69 | N1 | T0/T1 | Cell type not determined | 0 | ILC | 30 |
| 60-69 | N1 | T0/T1 | Cell type not determined | 0 | Other than ILC | 31 |
| 60-69 | N1 | T0/T1 | Cell type not determined | 1 | Other than ILC | 30 |
| 60-69 | N1 | T0/T1 | Cell type not determined | 2+ | Other than ILC | 32 |
| 60-69 | N1 | T2 | Well differentiated | 0 | ILC | 34 |
| 60-69 | N1 | T2 | Well differentiated | 0 | Other than ILC | 36 |
| 60-69 | N1 | T2 | Well differentiated | 1 | ILC | 33 |
| 60-69 | N1 | T2 | Well differentiated | 1 | Other than ILC | 35 |
| 60-69 | N1 | T2 | Well differentiated | 2+ | Other than ILC | 37 |
| 60-69 | N1 | T2 | Moderately differentiated | 0 | ILC | 38 |
| 60-69 | N1 | T2 | Moderately differentiated | 0 | Other than ILC | 40 |
| 60-69 | N1 | T2 | Moderately differentiated | 1 | ILC | 37 |
| 60-69 | N1 | T2 | Moderately differentiated | 1 | Other than ILC | 39 |
| 60-69 | N1 | T2 | Moderately differentiated | 2+ | Other than ILC | 41 |
| 60-69 | N1 | T2 | Poorly differentiated / Undifferentiated | 0 | ILC | 46 |
| 60-69 | N1 | T2 | Poorly differentiated / Undifferentiated | 0 | Other than ILC | 48 |
| 60-69 | N1 | T2 | Poorly differentiated / Undifferentiated | 1 | ILC | 45 |
| 60-69 | N1 | T2 | Poorly differentiated / Undifferentiated | 1 | Other than ILC | 47 |
| 60-69 | N1 | T2 | Poorly differentiated / Undifferentiated | 2+ | ILC | 47 |
| 60-69 | N1 | T2 | Cell type not determined | 0 | ILC | 36 |
| 60-69 | N1 | T2 | Cell type not determined | 0 | Other than ILC | 38 |
| 60-69 | N1 | T2 | Cell type not determined | 1 | ILC | 35 |
| 60-69 | N1 | T2 | Cell type not determined | 1 | Other than ILC | 37 |
| 60-69 | N1 | T3/T4 | Well differentiated | 0 | ILC | 46 |
| 60-69 | N1 | T3/T4 | Well differentiated | 0 | Other than ILC | 48 |
| 60-69 | N1 | T3/T4 | Well differentiated | 1 | ILC | 45 |
| 60-69 | N1 | T3/T4 | Well differentiated | 1 | Other than ILC | 47 |
| 60-69 | N1 | T3/T4 | Moderately differentiated | 0 | ILC | 50 |
| 60-69 | N1 | T3/T4 | Moderately differentiated | 0 | Other than ILC | 52 |
| 60-69 | N1 | T3/T4 | Moderately differentiated | 1 | Other than ILC | 51 |
| 60-69 | N1 | T3/T4 | Poorly differentiated / Undifferentiated | 0 | ILC | 58 |
| 60-69 | N1 | T3/T4 | Poorly differentiated / Undifferentiated | 0 | Other than ILC | 60 |
| 60-69 | N1 | T3/T4 | Cell type not determined | 0 | ILC | 48 |
| 60-69 | N1 | T3/T4 | Cell type not determined | 0 | Other than ILC | 50 |
| 60-69 | N2+ | T0/T1 | Well differentiated | 0 | ILC | 61 |
| 60-69 | N2+ | T0/T1 | Well differentiated | 0 | Other than ILC | 63 |
| 60-69 | N2+ | T0/T1 | Well differentiated | 2+ | ILC | 62 |
| 60-69 | N2+ | T0/T1 | Moderately differentiated | 0 | ILC | 65 |
| 60-69 | N2+ | T0/T1 | Moderately differentiated | 0 | Other than ILC | 67 |
| 60-69 | N2+ | T0/T1 | Poorly differentiated / Undifferentiated | 0 | Other than ILC | 73 |
| 60-69 | N2+ | T0/T1 | Poorly differentiated / Undifferentiated | 1 | Other than ILC | 72 |
| 60-69 | N2+ | T0/T1 | Cell type not determined | 0 | Other than ILC | 64 |
| 60-69 | N2+ | T2 | Well differentiated | 0 | ILC | 67 |
| 60-69 | N2+ | T2 | Well differentiated | 0 | Other than ILC | 69 |
| 60-69 | N2+ | T2 | Moderately differentiated | 0 | ILC | 71 |
| 60-69 | N2+ | T2 | Moderately differentiated | 0 | Other than ILC | 73 |
| 60-69 | N2+ | T2 | Moderately differentiated | 1 | ILC | 70 |
| 60-69 | N2+ | T2 | Moderately differentiated | 1 | Other than ILC | 71 |
| 60-69 | N2+ | T2 | Moderately differentiated | 2+ | ILC | 71 |
| 60-69 | N2+ | T2 | Moderately differentiated | 2+ | Other than ILC | 73 |
| 60-69 | N2+ | T2 | Poorly differentiated / Undifferentiated | 0 | ILC | 77 |
| 60-69 | N2+ | T2 | Poorly differentiated / Undifferentiated | 0 | Other than ILC | 79 |
| 60-69 | N2+ | T2 | Poorly differentiated / Undifferentiated | 1 | Other than ILC | 78 |
| 60-69 | N2+ | T3/T4 | Well differentiated | 0 | ILC | 77 |
| 60-69 | N2+ | T3/T4 | Moderately differentiated | 0 | ILC | 80 |
| 60-69 | N2+ | T3/T4 | Moderately differentiated | 0 | Other than ILC | 81 |
| 60-69 | N2+ | T3/T4 | Moderately differentiated | 1 | ILC | 79 |
| 60-69 | N2+ | T3/T4 | Moderately differentiated | 1 | Other than ILC | 80 |
| 60-69 | N2+ | T3/T4 | Moderately differentiated | 2+ | Other than ILC | 82 |
| 70-79 | N1mi | T0/T1 | Well differentiated | 0 | ILC | 9 |
| 70-79 | N1mi | T0/T1 | Well differentiated | 0 | Other than ILC | 9 |
| 70-79 | N1mi | T0/T1 | Well differentiated | 1 | ILC | 8 |
| 70-79 | N1mi | T0/T1 | Well differentiated | 1 | Other than ILC | 9 |
| 70-79 | N1mi | T0/T1 | Well differentiated | 2+ | Other than ILC | 9 |
| 70-79 | N1mi | T0/T1 | Moderately differentiated | 0 | ILC | 10 |
| 70-79 | N1mi | T0/T1 | Moderately differentiated | 0 | Other than ILC | 11 |
| 70-79 | N1mi | T0/T1 | Moderately differentiated | 1 | ILC | 9 |
| 70-79 | N1mi | T0/T1 | Moderately differentiated | 1 | Other than ILC | 10 |
| 70-79 | N1mi | T0/T1 | Moderately differentiated | 2+ | Other than ILC | 11 |
| 70-79 | N1mi | T0/T1 | Poorly differentiated / Undifferentiated | 0 | ILC | 13 |
| 70-79 | N1mi | T0/T1 | Poorly differentiated / Undifferentiated | 0 | Other than ILC | 14 |
| 70-79 | N1mi | T0/T1 | Poorly differentiated / Undifferentiated | 1 | Other than ILC | 14 |
| 70-79 | N1mi | T0/T1 | Poorly differentiated / Undifferentiated | 2+ | ILC | 14 |
| 70-79 | N1mi | T0/T1 | Poorly differentiated / Undifferentiated | 2+ | Other than ILC | 15 |
| 70-79 | N1mi | T0/T1 | Cell type not determined | 0 | ILC | 9 |
| 70-79 | N1mi | T0/T1 | Cell type not determined | 0 | Other than ILC | 10 |
| 70-79 | N1mi | T0/T1 | Cell type not determined | 1 | Other than ILC | 9 |
| 70-79 | N1mi | T2 | Well differentiated | 0 | ILC | 11 |
| 70-79 | N1mi | T2 | Well differentiated | 0 | Other than ILC | 12 |
| 70-79 | N1mi | T2 | Well differentiated | 1 | ILC | 11 |
| 70-79 | N1mi | T2 | Well differentiated | 1 | Other than ILC | 11 |
| 70-79 | N1mi | T2 | Well differentiated | 2+ | Other than ILC | 12 |
| 70-79 | N1mi | T2 | Moderately differentiated | 0 | ILC | 13 |
| 70-79 | N1mi | T2 | Moderately differentiated | 0 | Other than ILC | 14 |
| 70-79 | N1mi | T2 | Moderately differentiated | 1 | ILC | 12 |
| 70-79 | N1mi | T2 | Moderately differentiated | 1 | Other than ILC | 13 |
| 70-79 | N1mi | T2 | Moderately differentiated | 2+ | ILC | 13 |
| 70-79 | N1mi | T2 | Moderately differentiated | 2+ | Other than ILC | 14 |
| 70-79 | N1mi | T2 | Poorly differentiated / Undifferentiated | 0 | ILC | 17 |
| 70-79 | N1mi | T2 | Poorly differentiated / Undifferentiated | 0 | Other than ILC | 18 |
| 70-79 | N1mi | T2 | Poorly differentiated / Undifferentiated | 1 | Other than ILC | 17 |
| 70-79 | N1mi | T2 | Cell type not determined | 0 | ILC | 12 |
| 70-79 | N1mi | T2 | Cell type not determined | 1 | Other than ILC | 12 |
| 70-79 | N1mi | T3/T4 | Well differentiated | 0 | ILC | 17 |
| 70-79 | N1mi | T3/T4 | Well differentiated | 0 | Other than ILC | 18 |
| 70-79 | N1mi | T3/T4 | Moderately differentiated | 0 | ILC | 19 |
| 70-79 | N1mi | T3/T4 | Moderately differentiated | 0 | Other than ILC | 20 |
| 70-79 | N1mi | T3/T4 | Poorly differentiated / Undifferentiated | 0 | ILC | 25 |
| 70-79 | N1 | T0/T1 | Well differentiated | 0 | ILC | 17 |
| 70-79 | N1 | T0/T1 | Well differentiated | 0 | Other than ILC | 19 |
| 70-79 | N1 | T0/T1 | Well differentiated | 1 | Other than ILC | 18 |
| 70-79 | N1 | T0/T1 | Well differentiated | 2+ | Other than ILC | 19 |
| 70-79 | N1 | T0/T1 | Moderately differentiated | 0 | ILC | 20 |
| 70-79 | N1 | T0/T1 | Moderately differentiated | 0 | Other than ILC | 21 |
| 70-79 | N1 | T0/T1 | Moderately differentiated | 1 | ILC | 19 |
| 70-79 | N1 | T0/T1 | Moderately differentiated | 1 | Other than ILC | 20 |
| 70-79 | N1 | T0/T1 | Moderately differentiated | 2+ | ILC | 20 |
| 70-79 | N1 | T0/T1 | Moderately differentiated | 2+ | Other than ILC | 22 |
| 70-79 | N1 | T0/T1 | Poorly differentiated / Undifferentiated | 0 | ILC | 26 |
| 70-79 | N1 | T0/T1 | Poorly differentiated / Undifferentiated | 0 | Other than ILC | 27 |
| 70-79 | N1 | T0/T1 | Poorly differentiated / Undifferentiated | 1 | Other than ILC | 26 |
| 70-79 | N1 | T0/T1 | Cell type not determined | 0 | ILC | 18 |
| 70-79 | N1 | T0/T1 | Cell type not determined | 0 | Other than ILC | 20 |
| 70-79 | N1 | T0/T1 | Cell type not determined | 1 | ILC | 18 |
| 70-79 | N1 | T0/T1 | Cell type not determined | 1 | Other than ILC | 19 |
| 70-79 | N1 | T2 | Well differentiated | 0 | ILC | 22 |
| 70-79 | N1 | T2 | Well differentiated | 0 | Other than ILC | 23 |
| 70-79 | N1 | T2 | Well differentiated | 1 | ILC | 21 |
| 70-79 | N1 | T2 | Well differentiated | 1 | Other than ILC | 22 |
| 70-79 | N1 | T2 | Well differentiated | 2+ | ILC | 22 |
| 70-79 | N1 | T2 | Well differentiated | 2+ | Other than ILC | 24 |
| 70-79 | N1 | T2 | Moderately differentiated | 0 | ILC | 25 |
| 70-79 | N1 | T2 | Moderately differentiated | 0 | Other than ILC | 26 |
| 70-79 | N1 | T2 | Moderately differentiated | 1 | ILC | 24 |
| 70-79 | N1 | T2 | Moderately differentiated | 1 | Other than ILC | 25 |
| 70-79 | N1 | T2 | Moderately differentiated | 2+ | ILC | 25 |
| 70-79 | N1 | T2 | Moderately differentiated | 2+ | Other than ILC | 27 |
| 70-79 | N1 | T2 | Poorly differentiated / Undifferentiated | 0 | ILC | 31 |
| 70-79 | N1 | T2 | Poorly differentiated / Undifferentiated | 0 | Other than ILC | 33 |
| 70-79 | N1 | T2 | Poorly differentiated / Undifferentiated | 1 | ILC | 30 |
| 70-79 | N1 | T2 | Poorly differentiated / Undifferentiated | 1 | Other than ILC | 32 |
| 70-79 | N1 | T2 | Poorly differentiated / Undifferentiated | 2+ | Other than ILC | 34 |
| 70-79 | N1 | T2 | Cell type not determined | 0 | ILC | 23 |
| 70-79 | N1 | T2 | Cell type not determined | 0 | Other than ILC | 24 |
| 70-79 | N1 | T2 | Cell type not determined | 1 | Other than ILC | 24 |
| 70-79 | N1 | T3/T4 | Well differentiated | 0 | ILC | 31 |
| 70-79 | N1 | T3/T4 | Well differentiated | 0 | Other than ILC | 33 |
| 70-79 | N1 | T3/T4 | Well differentiated | 1 | ILC | 30 |
| 70-79 | N1 | T3/T4 | Well differentiated | 2+ | Other than ILC | 34 |
| 70-79 | N1 | T3/T4 | Moderately differentiated | 0 | ILC | 35 |
| 70-79 | N1 | T3/T4 | Moderately differentiated | 0 | Other than ILC | 37 |
| 70-79 | N1 | T3/T4 | Moderately differentiated | 1 | ILC | 34 |
| 70-79 | N1 | T3/T4 | Moderately differentiated | 1 | Other than ILC | 35 |
| 70-79 | N1 | T3/T4 | Moderately differentiated | 2+ | ILC | 35 |
| 70-79 | N1 | T3/T4 | Moderately differentiated | 2+ | Other than ILC | 37 |
| 70-79 | N1 | T3/T4 | Poorly differentiated / Undifferentiated | 0 | ILC | 43 |
| 70-79 | N1 | T3/T4 | Poorly differentiated / Undifferentiated | 0 | Other than ILC | 45 |
| 70-79 | N1 | T3/T4 | Poorly differentiated / Undifferentiated | 1 | Other than ILC | 43 |
| 70-79 | N1 | T3/T4 | Cell type not determined | 0 | ILC | 33 |
| 70-79 | N1 | T3/T4 | Cell type not determined | 0 | Other than ILC | 35 |
| 70-79 | N2+ | T0/T1 | Well differentiated | 0 | Other than ILC | 48 |
| 70-79 | N2+ | T0/T1 | Well differentiated | 1 | Other than ILC | 46 |
| 70-79 | N2+ | T0/T1 | Moderately differentiated | 0 | Other than ILC | 52 |
| 70-79 | N2+ | T0/T1 | Moderately differentiated | 1 | Other than ILC | 50 |
| 70-79 | N2+ | T0/T1 | Poorly differentiated / Undifferentiated | 0 | Other than ILC | 60 |
| 70-79 | N2+ | T0/T1 | Poorly differentiated / Undifferentiated | 2+ | Other than ILC | 60 |
| 70-79 | N2+ | T0/T1 | Cell type not determined | 0 | ILC | 47 |
| 70-79 | N2+ | T2 | Well differentiated | 0 | ILC | 53 |
| 70-79 | N2+ | T2 | Well differentiated | 0 | Other than ILC | 55 |
| 70-79 | N2+ | T2 | Well differentiated | 1 | ILC | 51 |
| 70-79 | N2+ | T2 | Well differentiated | 1 | Other than ILC | 53 |
| 70-79 | N2+ | T2 | Well differentiated | 2+ | ILC | 53 |
| 70-79 | N2+ | T2 | Moderately differentiated | 0 | ILC | 57 |
| 70-79 | N2+ | T2 | Moderately differentiated | 0 | Other than ILC | 59 |
| 70-79 | N2+ | T2 | Moderately differentiated | 1 | ILC | 55 |
| 70-79 | N2+ | T2 | Moderately differentiated | 1 | Other than ILC | 57 |
| 70-79 | N2+ | T2 | Poorly differentiated / Undifferentiated | 0 | Other than ILC | 66 |
| 70-79 | N2+ | T2 | Cell type not determined | 0 | ILC | 54 |
| 70-79 | N2+ | T2 | Cell type not determined | 0 | Other than ILC | 56 |
| 70-79 | N2+ | T2 | Cell type not determined | 1 | Other than ILC | 55 |
| 70-79 | N2+ | T3/T4 | Well differentiated | 0 | ILC | 64 |
| 70-79 | N2+ | T3/T4 | Well differentiated | 0 | Other than ILC | 66 |
| 70-79 | N2+ | T3/T4 | Moderately differentiated | 0 | ILC | 68 |
| 70-79 | N2+ | T3/T4 | Moderately differentiated | 0 | Other than ILC | 70 |
| 70-79 | N2+ | T3/T4 | Moderately differentiated | 1 | ILC | 67 |
| 70-79 | N2+ | T3/T4 | Moderately differentiated | 1 | Other than ILC | 69 |
| 70-79 | N2+ | T3/T4 | Moderately differentiated | 2+ | ILC | 69 |
| 70-79 | N2+ | T3/T4 | Poorly differentiated / Undifferentiated | 1 | ILC | 74 |
| 80+ | N1mi | T0/T1 | Well differentiated | 0 | ILC | 4 |
| 80+ | N1mi | T0/T1 | Well differentiated | 0 | Other than ILC | 5 |
| 80+ | N1mi | T0/T1 | Well differentiated | 1 | Other than ILC | 5 |
| 80+ | N1mi | T0/T1 | Moderately differentiated | 0 | ILC | 5 |
| 80+ | N1mi | T0/T1 | Moderately differentiated | 0 | Other than ILC | 6 |
| 80+ | N1mi | T0/T1 | Moderately differentiated | 1 | Other than ILC | 5 |
| 80+ | N1mi | T0/T1 | Moderately differentiated | 2+ | Other than ILC | 6 |
| 80+ | N1mi | T0/T1 | Poorly differentiated / Undifferentiated | 0 | Other than ILC | 8 |
| 80+ | N1mi | T2 | Moderately differentiated | 0 | Other than ILC | 7 |
| 80+ | N1mi | T2 | Moderately differentiated | 1 | Other than ILC | 7 |
| 80+ | N1mi | T2 | Moderately differentiated | 2+ | Other than ILC | 8 |
| 80+ | N1mi | T3/T4 | Well differentiated | 0 | Other than ILC | 10 |
| 80+ | N1mi | T3/T4 | Well differentiated | 1 | Other than ILC | 9 |
| 80+ | N1mi | T3/T4 | Moderately differentiated | 0 | ILC | 11 |
| 80+ | N1 | T0/T1 | Well differentiated | 0 | ILC | 10 |
| 80+ | N1 | T0/T1 | Well differentiated | 0 | Other than ILC | 10 |
| 80+ | N1 | T0/T1 | Well differentiated | 1 | Other than ILC | 10 |
| 80+ | N1 | T0/T1 | Well differentiated | 2+ | Other than ILC | 11 |
| 80+ | N1 | T0/T1 | Moderately differentiated | 0 | ILC | 11 |
| 80+ | N1 | T0/T1 | Moderately differentiated | 0 | Other than ILC | 12 |
| 80+ | N1 | T0/T1 | Moderately differentiated | 1 | Other than ILC | 11 |
| 80+ | N1 | T0/T1 | Moderately differentiated | 2+ | ILC | 11 |
| 80+ | N1 | T0/T1 | Poorly differentiated / Undifferentiated | 0 | Other than ILC | 16 |
| 80+ | N1 | T0/T1 | Poorly differentiated / Undifferentiated | 1 | Other than ILC | 15 |
| 80+ | N1 | T0/T1 | Cell type not determined | 0 | ILC | 10 |
| 80+ | N1 | T0/T1 | Cell type not determined | 0 | Other than ILC | 11 |
| 80+ | N1 | T2 | Well differentiated | 0 | ILC | 12 |
| 80+ | N1 | T2 | Well differentiated | 0 | Other than ILC | 13 |
| 80+ | N1 | T2 | Well differentiated | 1 | ILC | 12 |
| 80+ | N1 | T2 | Well differentiated | 1 | Other than ILC | 13 |
| 80+ | N1 | T2 | Well differentiated | 2+ | Other than ILC | 14 |
| 80+ | N1 | T2 | Moderately differentiated | 0 | ILC | 14 |
| 80+ | N1 | T2 | Moderately differentiated | 0 | Other than ILC | 15 |
| 80+ | N1 | T2 | Moderately differentiated | 1 | Other than ILC | 15 |
| 80+ | N1 | T2 | Moderately differentiated | 2+ | ILC | 15 |
| 80+ | N1 | T2 | Moderately differentiated | 2+ | Other than ILC | 16 |
| 80+ | N1 | T2 | Poorly differentiated / Undifferentiated | 0 | Other than ILC | 20 |
| 80+ | N1 | T2 | Poorly differentiated / Undifferentiated | 1 | Other than ILC | 19 |
| 80+ | N1 | T2 | Cell type not determined | 0 | Other than ILC | 14 |
| 80+ | N1 | T3/T4 | Moderately differentiated | 0 | ILC | 21 |
| 80+ | N1 | T3/T4 | Moderately differentiated | 0 | Other than ILC | 23 |
| 80+ | N1 | T3/T4 | Moderately differentiated | 1 | Other than ILC | 22 |
| 80+ | N1 | T3/T4 | Poorly differentiated / Undifferentiated | 0 | Other than ILC | 29 |
| 80+ | N1 | T3/T4 | Cell type not determined | 0 | ILC | 20 |
| 80+ | N2+ | T0/T1 | Cell type not determined | 0 | Other than ILC | 33 |
| 80+ | N2+ | T2 | Well differentiated | 0 | Other than ILC | 38 |
| 80+ | N2+ | T2 | Moderately differentiated | 0 | ILC | 40 |
| 80+ | N2+ | T2 | Moderately differentiated | 0 | Other than ILC | 41 |
| 80+ | N2+ | T2 | Moderately differentiated | 1 | Other than ILC | 40 |
| 80+ | N2+ | T2 | Poorly differentiated / Undifferentiated | 1 | Other than ILC | 48 |
| 80+ | N2+ | T2 | Cell type not determined | 0 | ILC | 37 |
| 80+ | N2+ | T3/T4 | Well differentiated | 0 | ILC | 48 |
| 80+ | N2+ | T3/T4 | Moderately differentiated | 0 | ILC | 52 |
| 80+ | N2+ | T3/T4 | Cell type not determined | 1 | Other than ILC | 50 |
